# MRI Study on Reversible and Irreversible Electroporation Induced Blood Brain Barrier Disruption

**DOI:** 10.1371/journal.pone.0042817

**Published:** 2012-08-10

**Authors:** Mohammad Hjouj, David Last, David Guez, Dianne Daniels, Shirley Sharabi, Jacob Lavee, Boris Rubinsky, Yael Mardor

**Affiliations:** 1 Center for Bioengineering in the Service of Humanity and Society, School of Computer Science and Engineering, Hebrew University, Jerusalem, Israel; 2 The Advanced Technology Center, Sheba Medical Center, Ramat-Gan, Israel; 3 Sackler School of Medicine, Tel-Aviv University, Tel-Aviv, Israel; 4 Cardiology Institute, Sheba Medical Center, Ramat-Gan, Israel; 5 Department of Mechanical Engineering, University of California, Berkeley, California, United States of America; Washington University, United States of America

## Abstract

Electroporation, is known to induce cell membrane permeabilization in the reversible (RE) mode and cell death in the irreversible (IRE) mode. Using an experimental system designed to produce a continuum of IRE followed by RE around a single electrode we used MRI to study the effects of electroporation on the brain. Fifty-four rats were injected with Gd-DOTA and treated with a G25 electrode implanted 5.5 mm deep into the striata. MRI was acquired immediately after treatment, 10 min, 20 min, 30 min, and up to three weeks following the treatment using: T1W, T2W, Gradient echo (GE), serial SPGR (DCE-MRI) with flip angles ranging over 5–25°, and diffusion-weighted MRI (DWMRI). Blood brain barrier (BBB) disruption was depicted as clear enhancement on T1W images. The average signal intensity in the regions of T1-enhancement, representing BBB disruption, increased from 1887±83 (arbitrary units) immediately post treatment to 2246±94 20 min post treatment, then reached a plateau towards the 30 min scan where it reached 2289±87. DWMRI at 30 min showed no significant effects. Early treatment effects and late irreversible damage were clearly depicted on T2W. The enhancing volume on T2W has increased by an average of 2.27±0.27 in the first 24–48 hours post treatment, suggesting an inflammatory tissue response. The permanent tissue damage, depicted as an enhancing region on T2W, 3 weeks post treatment, decreased to an average of 50±10% of the T2W enhancing volumes on the day of the treatment which was 33±5% of the BBB disruption volume. Permanent tissue damage was significantly smaller than the volume of BBB disruption, suggesting, that BBB disruption is associated with RE while tissue damage with IRE. These results demonstrate the feasibility of applying reversible and irreversible electroporation for transient BBB disruption or permanent damage, respectively, and applying MRI for planning/monitoring disruption volume/shape by optimizing electrode positions and treatment parameters.

## Introduction

Electroporation is a significant increase in the permeability of the cell membrane due to the formation of nanoscale defects in the membrane caused by certain electric fields across the cell [1], [2], [3]. As a function of the electrical parameters used, the cells can survive and the membrane can reseal resulting in reversible electroporation (RE), or the cells succumb to the electroporation and fail to reseal resulting in irreversible electroporation (IRE). Reversible electroporation is commonly used in molecular biology for introducing chemical species into cells, for which the cell membrane is normally impermeant to [4]. Irreversible electroporation delivered in a non-thermal mode - “Non thermal irreversible electroporation” (NTIRE) - is emerging as a highly promising modality for tissue ablation and treatment of tumors [5], [6], [7], [8], [9], [10], [11]. Unlike thermal ablation, NTIRE affects only the cell membrane, while preserving the extracellular scaffold. Consequently lumen structures such as blood vessels, bile ducts and intestines remain patent and regeneration can occur [6], [8], [12], [13], [14], [15]. The ability to treat tumors near large blood vessels has important applications in various organs such as the liver, pancreas and brain.

The group of Davalos has studied the use of NTIRE in the brain extensively, [16], [17] and has achieved good clinical results in the treatment of tumors in a canine brain [18]. Our group is particularly interested in developing means for enhanced drug delivery across the blood brain barrier and we use magnetic resonance imaging (MRI) as a research tool e.g. [19], [20]. Peripheral administration of therapeutic agents for the treatment of the central nerous system (CNS) pathologies is mostly inefficient due to poor penetration of most drugs across the blood brain barrier (BBB). Our goal in this study is to use MRI to advance understanding of the effects of reversible and irreversible electroporation on the blood brain barrier.

MRI was used in several important studies on electroporation. Some of the fundamental aspects of MRI imaging of electroporation were described in two papers of the late 1990’s [21], [22]. In an 1998 study, Sersa et al [21] have used albumin-(Gd-DTPA) contrast-enhanced magnetic resonance imaging to study blood flow in tumors treated with electroporation pulses. MRI was performed dynamically before and after intravenous administration of albumin 0.02 mmol Gd/kg. MRI images of tumors exposed to electric pulses showed no enhancement at 30 min after injection of albumin-(Gd-DTPA) leading to the conclusion that “electric pulses may, besides producing electroporation of cells, exert antitumor effectiveness by entrapping drugs within the tumors”. A 1999 study by Hannig et al. [22] acquired T_2_ -weighted and contrast enhanced T_1_- weighted images from a rat hind limb exposed to electric pulses. The authors found “a strong correlation between edematous tissue on (T_2_W) and areas of increased contrast agent distribution volume on (T_1_W) indicating the cell membrane permeabilizing nature of electroporation injury”.

Depiction of BBB opening has been widely applied to brain lesions in which significant BBB opening occurs, such as acute/subacute lesions of multiple sclerosis, brain tumors and metastases, inflammation, vascular disorders and head trauma [23]–[27]. These pathologies are depicted on contrast-enhanced MRI that is conventionally obtained by systemic administration of a Gd-based contrast agent, followed by a T1-weighted MRI. In this paper we apply contrast-enhanced T1-weighted MRI to demonstrate BBB disruption following electroporation treatments. T2-weighted MRI has been widely applied to determine the extent of brain tissue damage following disease and/or treatment [20], [28]–[31]. In this paper we apply T2-weighted MRI for assessing initial tissue response and permanent tissue damage following electroporation treatments.

Electrochemotherapy is an important application of reversible electroporation in which the electrical permeabilization of the cell membrane is used to introduce into cells drugs to which the cell membrane is otherwise impermeant [32]. A 2002 MRI study of electrochemotherapy in nude mice with a laryngeal tumor has shown that without the addition of bleomycin, the reversible electroporation pulsed electric fields had no effect on T1W images [33]. The addition of bleomycin has produced changes in T1, at 24 hours after the treatment. With respect to T2, there was an effect of reversible electroporation, which disappeared at 48 hours.

In vivo gene electrotransfer (ET) is a method of gene delivery that employs reversible electroporation to facilitate transfection of plasmid DNA into tissue [34]. A 2005 study performed on the rat tibialis muscle injected with a MRI contrast agent and a plasmid coding for luciferase has shown that the contrast agent produced an enhanced T1W image in the volume of tissue that was permeabilized and which expressed the luciferase [35]. There was quantitative agreement between the expression of the luciferase and the intracellular trapped contrast agent. T2W measurements provided good correlations to muscle necrosis. In continuation to [35], Bureau et al. [36], further study the use of reversible electroporation for gene transfection in the muscle with MRI. Gd-DOTA enhanced T1W in combination with histology and optical observations show that the concentration of gadolinium was significantly increased in the volume of tissue permeabilized with electric pulses. The permeabilized and the transfection level correlated for the set of all the conditions tested. However, no significant correlation was observed between Gd-DOTA concentration and transfection and therefore they conclude that “permeabilization is possibly not related to gene transfer but it indicates membrane modification related to transfection.” Inflammation was observed, with a maximum at day 3 after electroporation that was mostly reversed after 7 days. Aung et al [37], describe in a 2009 paper a very interesting strategy to monitor non-invasively electroporation induced gene transfection with MRI. They constructed a dual-reporter plasmid carrying a gene-encoding MRI reporter ferritin heavy chain and red fluorescent protein gene to visualize the transgene expression in a tumor model with optical and MRI measurements simultaneously. The plasmid-injected region showed both fluorescent emissions in optical imaging and detectably lowered signal on T2W MRI. The correlative immunohistological findings confirmed that both the reporter transgenes were co-expressed in this region. This strategy provides a platform for evaluating electroporation mediated gene therapy without administering a MRI contrast agent.

The development of NTIRE has generated an interest in imaging the procedure with various imaging modalities [8]. A 2010 study by Zhang et al. [38], was performed on the rat liver with T1- and T2-weighted images acquired before and immediately after application of the IRE pulses. MR imaging measurements were compared with numerical models and histologically confirmed ablation zones at necropsy. The comparison has shown that MR images permitted immediate depiction of IRE ablation zones that were hypointense on T1W images and hyperintense on T2W images. Hjouj and Rubinsky [39], used the potato as a model tissue to study various MRI sequences of NTIRE. The potato was used for NTIRE studies because cell damage is readily visible with optical means through a natural oxidation process of released intracellular enzymes (polyphenol oxidase) and the formation of brown-black melanins. MRI sequences of the treated area were taken at various times before and after NTIRE and compared with photographic images. A comparison was made between T1W, T2W, FLAIR and STIR MRI’s of NTIRE and photographic images. T1W and FLAIR sequences produce hyperintense images of the treated areas. In contrast, the signal was lost from the treated area and a hypointense image of the treated area was produced when a suppression technique, STIR, was used.

A fundamental study on MRI imaging of reversible electroporation in the brain was published recently in Mahmood et al. [40]. While emphasizing the value of MRI imaging to monitor reversible electroporation in the brain the study has proposed using diffusion-weighted magnetic resonance imaging (DW-MRI) as a method to monitor EP tissue using the concept of the apparent diffusion coefficient (ADC). The study examined the hypothesis that the plasma membrane permeabilization induced by EP changes the ADC. In vivo electroporation in rat brains, followed by DW-MRI found an electric pulse amplitude-dependent increase in the ADC following electroporation, indicating that “(1) DW-MRI is sensitive to the EP-induced changes and (2) the observed changes in ADC are indeed due to the applied electric field”.

In summary, all the studies on MRI of electroporation show that the effects of electroporation can be detected with various MRI sequences. The basic hypothesis which will be examined in this study is that in the brain non thermal irreversible electroporation (NTIRE) destroys the cells in the treated region while non-thermal reversible electroporation (NTRE) induces transient blood brain barrier (BBB) disruption around the ablated tissue. The study expands on the preliminary work in [41]. The hypothesis will be examined by imaging electroporated treated volume of brain tissue with gadolinium enhanced MRI and by comparing our experimental results with simulation results and the data from the group of Davalos.

## Materials and Methods

The study was specifically approved by and performed in accordance with the guidelines of The Animal Care and Use Committee of the Sheba Medical Center, which is approved by the Israeli authorities for animal experimentation.

We have developed an experimental strategy to study both irreversible and reversible electroporation in one experiment. The technology employs a single needle in the brain and a remote surface electrode. The nature of the electric field produced by this electrode configuration is that it is the highest at the electrode tissue interface and it tappers down away from the electrodes. Therefore, the electric fields around the electrodes will, if designed for that, start with NTIRE fields and gradually taper to non-thermal reversible electroporation (NTRE) fields. By imaging the treated volume of tissue with gadolinium enhanced MRI and by comparing our experimental results with simulation results and the data from the group of Davalos, we provide support for our hypothesis that NTIRE induces permanent brain tissue damage while NTRE induces transient BBB disruption.

Electroporation treatments were performed under full anesthesia using G25 electrodes implanted 5.5 mm deep into the rat striata. All rats were injected IP with Gd-DOTA (DOTAREM, 0.5 mmol/mL, Guerbet, France), 600 µl/kg, within 1–2 min prior to the electroporation treatment. MRI was acquired immediately post treatment and periodically thereafter.

### Animal Model

The study was performed using male Spring Dawly rats, 250–300 gr at the day of the electroporation treatment. Rats were anesthetized by intra muscular injections of 600 µL of 22.5 mg/mL ketamine and0.3% xylazine and placed in a stereotactic frame for electrode placement and treatment.

### Intracranial Electrode Placement

The bregma was identified through a midline scalp incision, and one 1 mm burr hole was drilled in the right or left region of the skull, 3 mm anterior and 2 mm lateral to the bregma. 25-gauge stainless-steel electrodes were placed stereotactically in the striatum at a depth of 5.5 mm. A second, large 4 cm by 8 cm flat electrode was pressed against the rat chest after applying conducting gel for better electric coupling. The electrodes were connected to the pulse generator. Control rats underwent similar procedures, including electrode implantation, without applying the electric pulses.

### Electroporation Treatment Protocol

Rats were treated using a conventional electroporator power supply (BTX 830; Harvard Apparatus, Holliston, MA). Voltages used in the experiments ranged from 250 V to 650 V, the number of pulses ranged from 50 to 90, the pulse duration ranged from 50 µs to 70 µs, and the pulse delivery frequency was 4 Hz.

### MRI Data Acquisition

All rats were scanned under full anesthesia using a clinical GE 1.5 T MRI system (Optima MR450w, General Electric, Milwaukee) with a clinical phased array knee coil and the following sequences: T1-weighted MRI, T2-weighted MRI, Gradient echo (GE), serial SPGR (DCE-MRI) with flip angles ranging over 5–25°, and diffusion-weighted MRI (DWMRI).

### Experiment #1: One Intracranial Electrode – Optimize Data Acquisition Timing Post Treatment

The goal of this experiment was to generate data on the BBB disruption immediately following the electroporation. The electroporation was performed using one intracranial electrode and another flat electrode pressed against the rat chest, as described earlier. Gd-DOTA was injected one to two minutes prior to the electroporation. The rats were then placed in the MR system for a continuous scan of 30 min. T1W MR Images were acquired immediately, 20 minutes and 30 minutes after electroporation. In addition, T1 relaxation values, reflecting the contrast agent concentrations, were calculated from DCE-MRI. Treatment parameters were set at 50 pulses of 70 µs duration at 4 Hz. Four rats were treated at 250 V, six rats at 350 V and four rats at 650 V. In each follow up we injected Gd-DOTA as described above.

### Experiment #2: One Intracranial Electrode - BBB Disruption and Permanent Tissue Damage Volumes

The second experiment was designed to study the correlation between BBB disruption volumes and permanent tissue damage. Thirty six rats were treated with one intracranial electrode, implanted in the rat striatum, and another flat electrode pressed against the rat chest, as described earlier. Treatment parameters were set at 90 pulses of 50 µs at 4 Hz. Eleven rats were treated at 250 V, six at 300 V, six at 350 V eight at 600 V and five at 650 V (Table 1). MRI followed the rats for three weeks. In the MRI follow up, Gd-DOTA was injected one to two minutes prior to the acquisition of the MR images. The volume of BBB disruption and tissue changes depicted on T2W MRI were correlated to the treatment parameters and time post treatment.

**Table 1:** Average volumes of BBB disruption and T2-enhancement.

Voltage [V]	# ofrats	T1 volume Day 0[mm^3^]	T2 volume Day 0[mm^3^]	T2/T1 volumeDay 0	T2 (Day 1–2)/T2(Day 0)	T2 (3 weeks)/T1(Day 0)
250	11	24.5±1.6	16.1±2.2	0.67±1.00	1.70±0.41	0.17±0.07
300	6	28.0±3.9	20.5±3.3	0.73±0.06	2.57±0.32	0.21±0.14
350	6	35.2±5.7	26.0±4.9	0.76±0.11	2.70±0.78	0.38±0.09
600	8	109.8±14.1	54.2±10.1	0.57±0.05	3.59±0.78	0.44±0.11
650	5	162.2±29.2	142.7±19.0	0.90±0.07	1.13±0.18	0.57±0.20

* Column 3: Enhancing volume on T1W images at the day of the treatment, representing the volume of BBB disruption (mean±SE).

** Column 4: Enhancing volume on T2W images at the day of the treatment, representing initial tissue response to treatment (mean±SE).

*** Column 5: Ratio of enhancing volume on T2W and T1W images on Day 0, showing that BBB disruption volume was always larger than tissue damage volume (mean±SE).

**** Column 6: Ratio of enhancing volume on T2W images 24–48 hours post treatment and T2W images on Day 0, showing the tissue response volume significantly increased in the first 2 days post treatment (mean±SE).

**** Column 7: Ratio of enhancing volume on T2-weighted MRI 3 weeks post treatment, representing the permanent tissue damage, and T1-weighted enhancing volume on Day 0, showing that BBB disruption was always larger than the permanent tissue damage (mean±SE).

### Experiment #3: One Intracranial Electrode – Delayed Contrast Injection

In an attempt to confirm the short term BBB disruption at low treatment voltage, 4 rats were treated with a set of 90 pulses of 50 µs at 4 Hz and at a low voltage of 250 V. The experimental setup was similar to that of experiments #1 and #2 except for the contrast agent injection protocol. Here the contrast agent was injected 20 min post electroporation, followed by MRI imaging.

### Mathematical Simulation

In order to determine the electric field generated near the energized electrode, a two-dimensional finite element model was implemented in the COMSOL software package (Comsol Multiphysics, v.4.2a; Stockholm, Sweden) as previously described [41], [42]. Numerical modeling was used to correlate the NTRE and NTIRE electric field threshold areas with MRI derived data on BBB disruption and tissue damage areas and to ensure that the temperature elevation is below the thermal damage threshold, as described in [5]. The rat head and chest were modeled as a 30 mm by 15 mm ellipse with one electrode inserted to a depth of 5.5 mm into the brain and the ground chest electrode at a distance of 25 mm, similar to the animal setup. The electrical boundary condition along the tissue in contact with the energized electrode was φ = Vo (electrode voltage) and φ = 0 at the ground electrode. The boundaries where the analyzed domain was not in contact with an electrode were treated as electrically isolative. Numerical simulations inputs were the pulse parameters (duration, voltage, number of repetitions, repetition time and intervals between pulses) and relevant physical properties of the tissue including: brain tissue electrical conductivity –0.35 S/m (stainless steel electrode conductivity 2.22E6 S/m). The mesh was built with 928 elements and the minimum element quality was set at 0.81.

## Results

### MRI Depiction of Electroporation Treatment Effects

**Figure 1:**
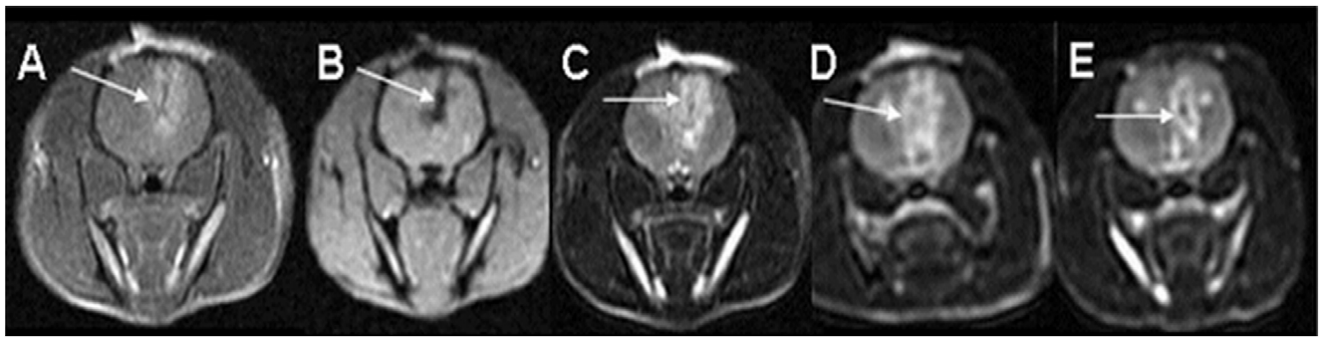
MRI sequences for depicting electroporation effects in the rat brain. T1W (A), Gradient-echo (B) and T2W (C–E) MRI of a rat treated with one intracranial electrode and another external flat electrode pressed against the rat chest. Treatment was performed with 50 pulses of 650 V, 70 µs duration and a frequency of 4 Hz. Significant BBB disruption is depicted as bright enhancement on the T1W images acquired 30 min after treatment (A). The GE image (B) shows signal void along the path of the electrode suggesting hemorrhage. T2W images depict tissue response to the treatment as bright enhancement (C–E). It can be seen that 1 day post treatment (D) the volume of tissue changes seems larger than on the day of the treatment (A), but then the volume is reduced by day 8 (E).

Clear enhancement on T1-weighted MRI was observed immediately after the electroporation treatment and continuously thereafter. Sequences acquired at 10 minute intervals following treatment show peak enhancement at 30 min post treatment. These effects are attributed to the BBB disruption. Early treatment effects and late irreversible damage were clearly depicted on T2-weighted MR images. The volumes of these effects of the electroporation treatment increased significantly in the first 24 hours post treatment and then decreases and stabilized by day 14 (Figure 1, Table 1). Hemorrhages depicted early on GE-MRI preceded permanent damage. DW-MRI acquired up to 30 min post treatment showed no significant effects.

### Experiment #1: One Intracranial Electrode – Optimize Data Acquisition Timing Post Treatment

Experiments were performed using one intracranial electrode and another flat electrode pressed against the rat chest. The experiments were designed to obtain optimal time for assessing BBB disruption at the day of the treatment. The average signal intensity in the regions of T1-enhancement, representing BBB disruption, increased from 1887±83 (arbitrary units) immediately post treatment to 2246±94 20 min post treatment, and then reached a plateau towards the 30 min scan where it reached 2289±87. Signal intensity of the contra-lateral regions remained constant (1507±39, 1502±38, 1500±36) suggesting no BBB disruption in the contra-lateral hemisphere.

**Figure 2:**
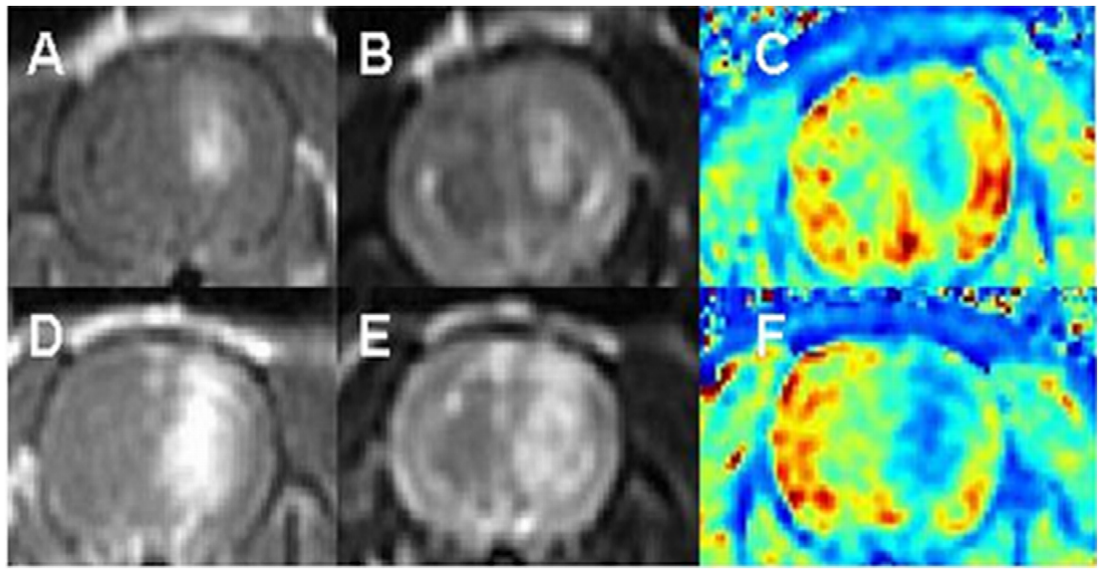
The effects of treatment voltage on BBB disruption volumes. T1W MRI (A, D), T2W MRI (B, E) and calculated T1 relaxation maps (C, F) of two rats treated with 50 pulses of 70 µs duration at 4 Hz. The MR images were acquired 30 min post treatment. Rat #1 (A–C) was treated at 350 V while rats #2 (D–F) was treated at 650 V. It can be seen that the BBB disruption volume (volume of enhancement on A and D) and the signal intensity on the T1W (enhancement intensity on A and D) are higher for rat #2 while the average T1-relaxation time (C and F) is lower.

The treatment voltages were found to correlate significantly with BBB disruption volumes, r^2^ = 0.70, p<0.002, with the mean signal intensity on the T1W MR images, r^2^ = 0.79, p<0.0001, and with T1 relaxation values (Figure 2), reflecting the contrast agent concentration in the brain, r^2^ = 0.68, p<0.0003.

**Figure 3:**
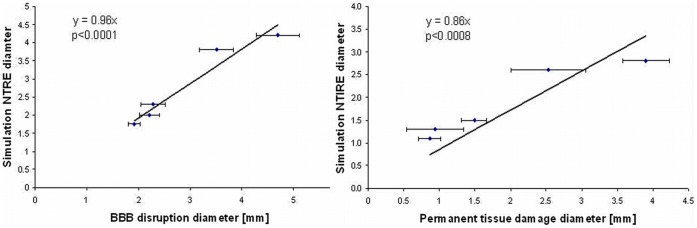
The correlation between calculated extent of NTRE and measured BBB disruption and between calculated extent of NTIRE and experimentally determined permanent tissue damage. The correlation between NTRE diameter, calculated from the simulation results, versus BBB disruption diameter, calculated from the rat MR data acquired at the day of the treatment, was found significant (A), suggesting that BBB disruption may be induced by NTRE, formed at electric fields above 330 V/cm. The correlation between NTIRE diameter, calculated using the simulation program, versus permanent tissue damage diameter, calculated from the rat MR data acquired 3 weeks post treatment, was found significant as well (B), suggesting that permanent tissue damage may be induced by NTIRE, formed at electric fields above 500 V/cm.

T1 signal intensity was highly correlated with T1 relaxation, as expected: r^2^ = 0.53, p<0.003 immediately post treatment and r^2^ = 0.81, p<0.0001 at 20 min post treatment. This correlation was best at 30 min post treatment: r^2^ = 0.88, p<0.0001.

There was no correlation between any of the measurable parameters calculated in the treatment region versus those calculated from contra-lateral brain regions.

**Figure 4:**
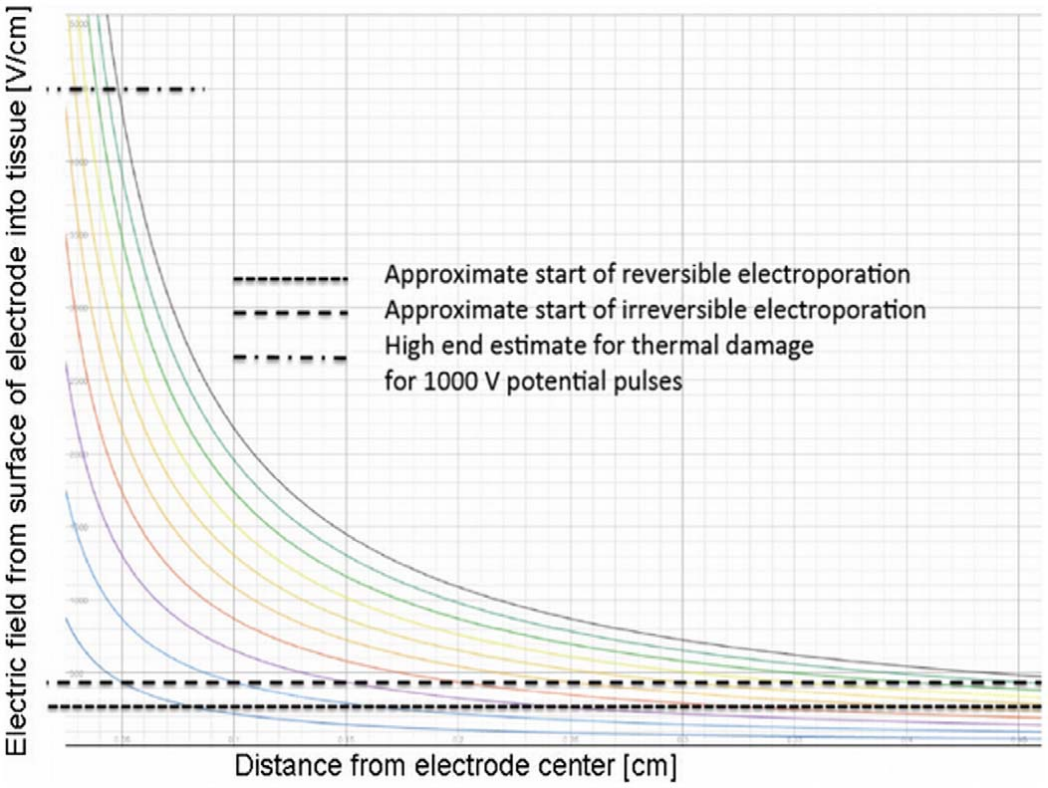
Electric field as a function of distance from center of electrode, calculated from a close form solution. The curved lines represent electric field for electrode voltages from 100 V (lower one) in increments of 100 V.

### Experiment #2: One Intracranial Electrode - BBB Disruption and Permanent Tissue Damage Volumes

These experiments were designed to study the correlation between treatment parameters, BBB disruption volumes and permanent tissue damage. All rats were treated with 90 pulses of 50 µs at 4 Hz. Average BBB disruption volumes and T2-enhancement volumes for each treatment voltage are listed in Table 1.

BBB disruption volumes were found to correlate significantly with the treatment voltage: r^2^ = 0.77, p<0.0001, suggesting larger BBB disruption volumes for higher treatment voltage values. The permanent tissue damage, depicted as enhancing regions on T2W MRI 3 weeks post treatment was also found to correlate significantly with the treatment voltage, r^2^ = 0.67, p<0.0001.

The enhancing volume on T2W MRI has increased by an average of 2.27±0.27 in the first 24–48 hours post treatment, suggesting an inflammatory tissue response and then decreased by day 8. There was no significant change in the T2 enhancing region from day 8 till the end of the follow-up, 3 weeks post treatment (p<0.55, Wilcoxon matched-pairs signed-ranks test), suggesting permanent tissue damage. The permanent tissue damage, calculated from the enhancing region on T2W 3 weeks post treatment, decreased to an average of 50±10% of the T2 enhancing volumes on the day of the treatment. Significant correlation, r^2^ = 0.62; p<0.0001, was also found between BBB disruption volumes, depicted as enhancing regions on T1W images acquired 30 min post treatment and later permanent damage, depicted as bright regions on T2W images acquired 3 weeks post treatment. This correlation implies that larger tissue damage volumes follow larger disruption volumes. The average volume of permanent tissue damage was only 33±5% of the BBB disruption volume on day 0. Thus, the permanent tissue damage was significantly smaller than the volume of BBB disruption for all rats. BBB disruption was still noticed in rats with significant tissue damage 3 days post treatment but reversed by day 8 in most rats.

### Experiment #3: One Intracranial Electrode – Delayed Contrast Injection

In experiment #1 it was found that BBB disruption increased significantly up to 20 min post treatment and then reached a plateau towards 30 min post treatment. In an attempt to confirm the short term BBB disruption at low treatment voltages, 3 additional rats were treated at 250 V with a delayed contrast injection, applied 20 min post treatment. T1W images acquired immediately post contrast injection showed no enhancement in the treated region even though T2W images depicted similar enhancing regions as in our previous experiments (10.6±4.2). Images acquired 20 min later showed minimal enhancement of 1.6±1.8 mm^3^ in volume, significantly smaller that the volumes of BBB disruption depicted when injecting the contrast agent prior to treatment (24.5±1.6 mm^3^). This enhancement was cleared by 40 min after contrast injection, when no enhancement was detected.

### Comparison with Simulation

In an attempt to study the mechanism of action causing BBB disruption and tissue damage following electroporation treatments, the results of experiment #2 were compared with the results of the simulation program. The nature of the electric field produce by electrodes is that it is the highest at the electrode tissue interface and it tappers down away from the electrodes.

The parameters used in the simulation program were based on tissue properties from the literature. The extent of the region experiencing reversible electroporation and irreversible electroporation were assessed from the simulation assuming that NTIRE occurs above 500 V/cm and NTRE above 330 V/cm [16].

BBB disruption diameter, calculated from the enhancing region on T1W MR Images on the day of the treatment, was found to correlate significantly with the diameter of NTRE calculated from the simulation (Figure 3A). The slope of the linear regression (forced to pass through (0,0)) shows that these parameters have similar values up to 4%. Permanent tissue damage diameter, calculated from the enhancing region on T2W MRI acquired 3 weeks post treatment, was found to correlate significantly with the diameter of NTIRE calculated from the simulation (Figure 3B). The calculated slope shows a discrepancy of ∼14% between the rat data and the simulation. The experimental configuration lends itself to an approximate close form solution in one-dimensional cylindrical coordinates around the electrode. A solution for the electric field equation as well as for the heat transfer equation ignoring blood flow effects is shown in Figure 4. The figure shows the calculated electric fields in tissue around the electrode for various voltages on the electrode. The fields have an ln(r), (r-radius), decay. The horizontal lines represent, from bottom to top – reversible electroporation, irreversible electroporation and thermal damage. The thermal damage was assessed as the condition in which a temperature of 50^o^ C is reached for a voltage of 1000 V on the electrode in the absence of blood flow. This serves therefore as an upper limit to the possible extent of thermal damage in tissue due to electroporation.

## Discussion

Our study was designed to assess the feasibility of applying electroporation for inducing temporary BBB disruption to enable efficient drug delivery into the brain. Once confirmed, this methodology may be applied for various CNS pathologies in which delivery of therapeutic doses across the BBB is currently unattainable.

In the case of primary brain tumors, which are infiltrative by nature, the optimal treatment should provide focal damage to the tumor mass combined with BBB disruption (enabling efficient delivery of systemically administered therapeutic agents) to the surrounding infiltrating zone. Electroporation has been previously shown to enable intracellular uptake of bleomycin (otherwise impermeable to the cell membrane) when applied in the tumor, thus enabling increased cell kill [33]. The mode of treatment presented in this paper should enable, in parallel to focal destruction of tumor mass, treatment of infiltrating tumor cells while sparing functioning brain tissue. Electroporation is ideal for such combined treatment by inducing permanent tissue damage in the vicinity of the intracranial electrode with NTIRE, where the electric fields are high and surrounding BBB disruption with NTRE for efficient drug delivery, at larger distances from the electrode, where the electric fields are lower. Assuming one treatment may not be sufficient, the intracranial electrode may be placed in the tumor for several weeks/months for repeated treatment sessions.

In our experiment, we found a significant correlation between the applied voltage and the volume of tissue damage, in concordance with a recent normal brain canine study [17]. A significant correlation was also determined between the treatment voltage and the volume of BBB disruption.

It is important to note that using our treatment parameters, BBB disruption was significantly correlated with later volume of tissue damage and in all cases depicted a larger volume than the final damage. These results imply that contrast-enhanced T1W MRI may be used for treatment monitoring in the case of brain electroporation where preservation of healthy tissue is crucial. Still, one has to take into account that tissue response is time dependent. Our data shows that using our treatment protocol the tissue response volume increased by a factor of 2.5 in the first 24–48 hours, and only later decreased to the final damage volume. This effect should be taken into consideration when planning intracranial treatments. The effect of initial increased tissue response may be explained by inflammatory tissue response to the treatment, in accord with previous observations [7]. This may also explain the fact that rats with significant permanent tissue damage showed residual BBB disruption several days post treatment.

The fact that BBB disruption volumes were always larger than the final tissue damage, also demonstrates the feasibility of applying the combined treatment, systemic chemo with local electroporation, for destruction of tumoral tissue by irreversible electroporation together with efficient chemotherapy, delivered to the surrounding tissue by temporary BBB disruption.

The high correlation between the signal intensity on T1W images calculated in experiment #3 and the T1 relaxation values, suggests that T1W images may be sufficient for assessing the level of BBB disruption in the range of parameters studied in our experiment with no need for longer measurements of T1 relaxation values.

Our basic hypothesis, presented in the Introduction section, was that NTRE induces transient BBB disruption while NTIRE induces irreversible tissue damage. The significant correlation found between the simulation results and our rat data suggest that indeed NTRE induces transient BBB disruption, with an electric field threshold of 330 V/cm with the single intracranial electrode setup, while NTIRE induces permanent tissue damage, with a threshold of 500 V/cm. The results of the histological evaluation were found consistent with this hypothesis. In addition, the results of the delayed contrast injection experiment, showing significantly less enhancing volumes, are consistent with this approach as well, since the effect of NTRE is expected to last for minutes only [2].

There was a large difference between the measured variables at 600 and 650 V (Table 1). A possible hypothesis is that it may be related to the change in electrical properties of tissue during electroporation. The permeabilization of the cell membrane by electroporation changes the electrical properties of tissue [43]. Still, these changes in electrical properties among different electroporation parameters are not linear, as the entire process of electroporation operates within electric fields thresholds. Nevertheless, this observation warrants further study. Since electroporation is expected to overall increase the conductivity of the tissue, regions on the border between RE and IRE may turn from RE into IRE, thus increasing the region of permanent tissue damage. This increase in IRE volume is expected to be more pronounced at high voltages, where the original IRE diameter is large. Since the change in conductivity was not included in the simulation, it may explain the weaker agreement between the simulation results and the permanent damage volumes (in contrast to the higher agreement with BBB disruption volumes), especially those obtained at 600–650 V which may be more affected by changes in electrical properties of tissues (Figure 3). Another possible explanation is from Figure 4. It is seen that the upper limit of thermal damage is within the range of electrode voltages of about 600–700 V and higher. Therefore, it may be possible that for these electrode voltages thermal damage begins to occur adjacent to the electrode during the application of the electroporation pulses. Thermal damage could substantially affect the electrical conductivity of the tissue and the overall behavior, resulting in the change in parameters between the 600 and 650 V potential on the electrode.

The blood–brain barrier (BBB) is a separation between the peripheral circulation and the central nervous system (CNS). Anatomically the BBB is composed of high density cells, the cerebral microvasular endothelium and the presence of tight cell to cell junctions (TJ) much more than endothelial cells in capillaries elsewhere in the body that restrict the diffusion of microscopic objects (e.g. bacteria) and large or hydrophilic molecules into the cerebrospinal fluid (CSF), while allowing the diffusion of small hydrophobic molecules (O_2_, CO_2_, hormones). Astrocyte cell projections surround the endothelial cells of the BBB, providing biochemical support to those cells. Cells of the barrier actively transport metabolic products such as glucose across the barrier with specific proteins [44]. As a result of BBB neuroprotective role, the delivery of many agents to the brain restricted. Molecules and genes that might be effective in diagnosis and therapy do not cross the BBB in adequate amounts.

Mechanisms for the delivery of diagnostic and therapeutic agents through the BBB involve going either “through” or “behind” the BBB, such as disruption by osmotic means; by localized exposure to high-intensity focused ultrasound (HIFU) [45] the use of endogenous transport systems, intracerebral implantation (such as with needles) and convection-enhanced distribution; and recently nanotechnology [46], [47].

The results of this study, show that electroporation has the ability to breach the BBB in a controlled way. The results suggest that in treating of brain tumors with non-thermal irreversible electroporation it should be possible to take advantage of the fact that NTRE affected volume extends beyond the NTIRE affected volume and that it breaches the BBB. Consequently it should be possible to more precisely target brain tissue ablation by treating the core of the tumor with NTIRE, while injecting drugs or genes to be incorporated in the surrounding volume of tissue in which NTRE yields a temporary breach of the BBB.

A particular additional hypothesis presents itself based on these studies and the paper of Lopez-Quentrero et al. [48]. They have found in work with cells that fields lower than those required for electroporation can also breach the BBB, through a mechanims that is not yet understood. The resolution of our experiments is not sufficient to verify this hypothesis, except that our observations show that the region in which the BBB is breached extends well beyond the NTIRE treated region to much lower electric fields. It is continuous and obviously encompasses also the NTRE region. Therefore the conclusion that NTRE breaches the BBB is valid. However, we cannot determine at this stage if the BBB breached region includes also regions substantially beyond NTRE with fields as those in [48]. This is a valuable area of research that should be explored in further studies.

### Conclusions

Our study applying non thermal reversible electroporation and non thermal irreversible electroporation electric fields to the rat brain demonstrates the feasibility of applying electroporation for significant and transient BBB disruption with and without permanent tissue damage, under real-time MR treatment monitoring and with late MR monitoring of treatment effects. Significant correlation was found between treatment voltage, extent of NTIRE and later volume of tissue damage. Significant correlation was found between treatment voltage extent of NTRE and BBB disruption volume. BBB disruption volume was significantly correlated with later volume of tissue damage and in all cases depicted a larger volume than the final damage. These results imply that MRI may be used for treatment monitoring of brain electroporation where preservation of healthy tissue is crucial. Furthermore, electroporation may be applied for a combined treatment of systemic chemo + local electroporation, for destruction of brain tumors tissue by IRE, while chemotherapy is efficiently delivered to the surrounding infiltrated tissue due to the larger coverage of temporary BBB disruption.
